# N-Acetylcysteine as Modulator of the Essential Trace Elements Copper and Zinc

**DOI:** 10.3390/antiox9111117

**Published:** 2020-11-12

**Authors:** Theresa Wolfram, Maria Schwarz, Michaela Reuß, Kristina Lossow, Mario Ost, Susanne Klaus, Tanja Schwerdtle, Anna P. Kipp

**Affiliations:** 1 Department of Molecular Nutritional Physiology, Institute of Nutritional Sciences, Friedrich Schiller University Jena, 07743 Jena, Germany; theresa.wolfram@uni-jena.de (T.W.); schwarz.maria@uni-jena.de (M.S.); michaelareuss@yahoo.de (M.R.); kristina.lossow@uni-jena.de (K.L.); 2 TraceAge-DFG Research Unit on Interactions of Essential Trace Elements in Healthy and Diseased Elderly, D-13353 Potsdam-Berlin-Jena-Wuppertal, Germany; tanja.schwerdtle@uni-potsdam.de; 3 German Institute of Human Nutrition, 14558 Nuthetal, Germany; mario.ost@dife.de (M.O.); klaus@dife.de (S.K.); 4 Department of Food Chemistry, Institute of Nutritional Science, University of Potsdam, 14558 Nuthetal, Germany; 5 German Federal Institute for Risk Assessment (BfR), 10589 Berlin, Germany

**Keywords:** N-acetylcysteine, copper, zinc, glutathione, Nrf2

## Abstract

N-acetylcysteine (NAC) is a frequently prescribed drug and known for its metal chelating capability. However, to date it is not well characterized whether NAC intake affects the homeostasis of essential trace elements. As a precursor of glutathione (GSH), NAC also has the potential to modulate the cellular redox homeostasis. Thus, we aimed to analyze effects of acute and chronic NAC treatment on the homeostasis of copper (Cu) and zinc (Zn) and on the activity of the redox-sensitive transcription factor Nrf2. Cells were exposed to 1 mM NAC and were co-treated with 50 μM Cu or Zn. We showed that NAC treatment reduced the cellular concentration of Zn and Cu. In addition, NAC inhibited the Zn-induced Nrf2 activation and limited the concomitant upregulation of cellular GSH concentrations. In contrast, mice chronically received NAC via drinking water (1 g NAC/100 mL). Cu and Zn concentrations were decreased in liver and spleen. In the duodenum, NQO1, TXNRD, and SOD activities were upregulated by NAC. All of them can be induced by Nrf2, thus indicating a putative Nrf2 activation. Overall, NAC modulates the homeostasis of Cu and Zn both in vitro and in vivo and accordingly affects the cellular redox balance.

## 1. Introduction

N-acetylcysteine (NAC) is a derivative of the amino acid cysteine which contains a reactive thiol group that participates in redox reactions. Oxidation of cysteine gives rise to the disulfide cystine. The cysteine/cystine redox couple is the most abundant one in plasma while glutathione (GSH) and glutathione disulfide (GSSG) are most important within cells. GSH is a tripeptide consisting of glutamate, cysteine, and glycine, in which the central cysteine is relatively protected from spontaneous oxidation. Cysteine is usually the rate-limiting amino acid for GSH synthesis [1]. Dietary cysteine needs to be actively absorbed in the intestine, while oral NAC can enter the intestinal epithelium by passive diffusion [2]. Thus, NAC is an interesting source to fuel the cellular cysteine pool and GSH synthesis modulating the cellular redox homeostasis and the redox capacity of whole organisms. The master regulator of the cellular redox balance is the transcription factor Nrf2 (NF-E2-related factor 2) which induces target genes involved in cytoprotection such as thioredoxin reductase 1 (TXNRD1) or NAD(P)H quinone oxidoreductase 1 (NQO1). In addition, Nrf2 enhances the de novo GSH synthesis in response to oxidative insults [3,4]. Under basal conditions, Nrf2 is bound to Keap1 resulting in the continuous degradation of Nrf2 via the proteasome [5,6]. However, upon exposure to a multitude of Nrf2 inducers, the degradation of Nrf2 is repressed, which leads to its accumulation and nuclear translocation [7,8]. In different experimental setups, the elimination of H_2_O_2_ by NAC suppressed Nrf2 activation [9,10]. Vice versa, redox-active, essential trace elements such as copper (Cu) and zinc (Zn) can enhance Nrf2 signaling [11,12]. Besides this, Cu and Zn are important constituents of enzymes to coordinate their structural or catalytic properties. For example, this is the case for the antioxidant enzyme Cu/Zn superoxide dismutase (SOD1).

Besides its antioxidant effects, NAC has been shown to have metal-chelating capabilities. Chelating agents are defined as compounds which are able to form complexes with metal ions and thus reduce their toxicity. The chelating capacity of NAC is modulated by its thiol group in combination with the carboxyl group. Therefore, NAC is used to treat acute heavy metal poisoning—e.g., against mercury, cadmium, or arsenic intoxication [13]. Even though this function is well established, there is only limited information on how NAC affects essential metals such as Zn and Cu. In a human intervention study, NAC (200 mg three times a day) application to ten healthy volunteers for 2 weeks did not affect plasma levels of calcium, magnesium, iron, Zn, and Cu [14]. As NAC is frequently prescribed as mucolytic drug [15]—e.g., as cold medication—a putative link between NAC intake and the homeostasis of essential trace elements needs to be studied in more detail because especially Zn is an important modulator of the immune response [16]. Based on this, we aimed to use cell culture and mouse models to analyze short-term and chronic NAC effects on Cu and Zn concentrations within cells and organs such as liver (important for trace element homeostasis), duodenum (important for trace element absorption), and spleen (organ of the immune system). We could show that Cu and Zn concentrations were reduced both in vitro and in vivo upon NAC treatment. As both NAC and free Cu and Zn ions can modulate the cellular redox homeostasis, we analyzed the activity of the redox-sensitive transcription factor Nrf2 as stable read-out for redox homeostasis. The enzyme activities of Nrf2-regulated genes were enhanced in the duodenum of mice after chronic NAC supply, while NAC inhibited the Zn-induced nuclear translocation of Nrf2 after short-term treatment in cell culture.

## 2. Materials and Methods

### 2.1. Cell Culture

The human hepatocellular carcinoma cell line HepG2 (ATCC^®^ HB-8065™), the colorectal adenocarcinoma cell line HT-29 (ATCC^®^ HTB-38^TM^), and the acute monocytic leukemia cell line THP-1 (ATCC^®^ TIB-202™) were cultured in Roswell Park Memorial Institute 1640 media (RPMI; ThermoFisher Scientific, Waltham, MA, USA) with 10% (*v/v*) fetal calf serum (FCS, Sigma-Aldrich, Steinheim, Germany), 1% (*v/v*) penicillin-streptomycin (ThermoFisher Scientific), and 1% GlutaMAX (ThermoFisher Scientific) at 37 °C in a 5% CO_2_ atmosphere. For experiments, the cells were seeded for 24 h and accordingly were treated with or without 1 mM NAC (Sigma-Aldrich) dissolved in culture medium without FCS. In parallel, copper sulfate (CuSO_4_) (Sigma-Aldrich/Merck) and zinc sulfate (ZnSO_4_) (Sigma-Aldrich/Merck) were added in a final concentration of 50 µM for 6 h. The concentrations of Cu and Zn were chosen based on previous experiments [17]. Cells were harvest 30 h after seeding.

### 2.2. Cell Viability Assays

The cell viability was detected by the methylthiazolyldiphenyl-tetrazoliumbromid (MTT, Sigma-Aldrich) reduction capacity and the cell number. For the cell number, cells were dissolved in phosphate-buffered saline (PBS, 140 mM sodium chloride, 10 mM disodium hydrogen phosphate, and 2.99 mM potassium dihydrogen phosphate (Carl Roth, Karlsruhe, Germany), pH 7.4). The cell number was determined using the Vi-Cell XR Cell Viability Analyzer (Beckman Coulter, Brea, CA, USA) with 0.4% trypan blue (Sigma-Aldrich) for staining dead cells. For the MTT reduction capacity, the medium was removed and FCS-free medium containing 0.5 mg/mL MTT was added to the cells. After 60 min of incubation, the medium was discarded and resulting formazan crystals were lysed in dimethyl sulfoxide (DMSO, Carl Roth) by a ten-minute shaking step at 600 rpm. Accordingly, the obtained formazan was measured photometrically at 550 nm with 690 nm as reference wavelength, by using a microplate reader (Synergy H1, BioTek, Bad Friedrichshall, Germany). All measurements were performed in triplicates using 96-well plates.

### 2.3. Animal Experiment

Male C57BL/6Jrj mice were housed on a 12:12 h light:dark schedule with food and tap water ad libitum. The mouse strain was chosen to allow for comparison with previous results [17]. A commercially available chow diet (V1534, Ssniff, Soest, Germany) with 97 and 8.8 mg/kg Zn and Cu, respectively, was used. After weaning at the age of four weeks, mice were assigned to the NAC-treated or the control group. The former received NAC via drinking water (1 g/100 mL), while the latter only received water. The NAC solution was refreshed every second day. At the age of 24 weeks, mice were anesthetized with isoflurane (Cp-pharma, Burgdorf, Germany) and blood was collected by cardiac puncture. Organs were surgically dissected and immediately frozen. All animal procedures were approved and conducted following national guidelines of the Ministry of Environment, Health and Consumer Protection of the federal state of Brandenburg (Germany, 2347-44-2017) and institutional guidelines of the German Institute of Human Nutrition Potsdam-Rehbruecke.

### 2.4. Enzyme Activities

For enzyme activities, cells and mouse tissues (duodenum, liver, spleen) were homogenized with a TissueLyser II (Qiagen, Hilden, Germany) in Tris buffer (100 mM Tris (Carl Roth), 300 mM KCl (Applichem, Darmstadt, Germany), pH 7.6 with 0.1% (*v/v*) Triton X-100 (Serva, Heidelberg, Germany), and 0.1% (*v/v*) protease inhibitor (Merck/Millipore, Burlington, MA, USA)) two times for 60 s at 30 Hz. To remove the cellular debris, the samples were centrifuged for 10 min at 4 °C and 15,000× *g*. The protein concentration was determined by Bradford analysis (Bio-Rad Laboratories, Munich, Germany). The protein lysates were used for NQO1, SOD, TXNRD, and glutathione peroxidase (GPX) activity measurements. All enzyme activities were measured photometrically using a microplate reader (Synergy H1) and were performed in triplicates using 96-well plates. The enzyme activity was normalized to the protein content of the samples. The methods for NQO1 [18], total TXNRD, and GPX [19] activities have been described previously. Briefly, NQO1 catalyzes the reduction of menadione to menadiol which in turn reduces MTT to its water-soluble formazan. TXNRDs convert 5-5′-dithiobis (2-nitrobenzoic acid) (DTNB) to 2-nitro-5-thiobenzoic acid (TNB) under NADPH consumption. GPX activity was measured in a NADPH-consuming glutathione reductase (GR)-coupled test using H_2_O_2_ as substrate. For SOD activity measurements, superoxide anion radicals were produced by 1,2,3-benzenetriol (pyrogallol) autoxidation [20]. One unit of SOD activity corresponded to a 50% inhibition of pyrogallol autoxidation [21].

### 2.5. Western Blots of Nuclear Lysates

For Western blot analysis, the nuclear lysates of HepG2 cells were used after 4 h of NAC, Zn, and Cu treatments. The cells were scratched from culture dishes with lysis buffer I (10 mM Hepes (Carl Roth); 1.5 mM MgCl_2_ (Carl Roth); 10 mM KCl (Applichem); 0.5 mM Dithiothreitol (DTT; Merck); 0.5 mM phenylmethylsulfonyl fluoride (PMSF; Carl Roth), and 0.1% (*v/v*) NP-40 Alternative (Merck); pH 7.9) and were incubated 10 min at room temperature (RT) under shaking. After a centrifugation step for 1 min (6800× *g*; 4 °C), the cell pellets were lysed in lysis buffer II (40 mM Hepes; 400 mM KCl; 10% glycerol (Carl Roth); 1 mM DTT, and 0.1 mM PMSF; pH 7.9) and 294 mM NaCl for 30 min (4 °C), followed by a 30 min centrifugation step (20,000× *g*; 4 °C). The supernatant containing the nuclear lysates was used for Bradford analysis (Bio-Rad) and Western Blot. SDS polyacrylamide gel electrophoresis was followed by immunoblotting of proteins to nitrocellulose membranes. After immunoblotting, membranes were gently shaken for 2 min in Ponceau S solution (0.2% (*w/v*) Ponceau S (Carl Roth) with 3% (*w/v*) trichloroacetic acid (Carl Roth)) and bands were recorded by ChemiDoc^TM^ MP Imaging System (Bio-Rad). Subsequently, membranes were blocked in 5% (*w/v*) non-fat dry milk in Tris-buffered saline containing 0.1% (*v/v*) Tween 20 (T-TBS) for 1 h at RT. The membranes were incubated with the following primary antibody overnight at 4 °C: rabbit anti-Nrf2 (1:1000; 12,721, Cell Signaling, Danvers, MA, USA). Horseradish peroxidase-coupled goat anti-rabbit IgG (1:50,000; 7074 S, Cell Signaling) served as a secondary antibody. For detection of protein bands, SuperSignal^TM^ West Dura (ThermoFisher Scientific) was used, and band intensities were densitometrically quantified by a ChemiDoc^TM^ MP Imaging System. Finally, target protein expression was normalized to Ponceau staining.

### 2.6. Total Reflection X-ray Fluorescence (TXRF) Spectroscopy

Trace elements can be analyzed using X-rays, generated by a molybdenum tube using the S2 Picofox™ (Bruker Nano GmbH, Berlin, Germany). Prior to measurement, mouse tissue samples were digested, whereas cell lysates were analyzed without further digestion. Briefly, 50 mg of tissue samples was weighted into microwave vessels and 830 µL ultrapure water, 900 µL 69% HNO_3_ (*v/v*) (Suprapure^®^, Merck/Millipore), 250 µL 30% H_2_O_2_ (*v/v*) (Sigma-Aldrich/Merck), and 20 µL of 200 mg/L yttrium-solution (Merck/Millipore) as digestion control were added. The samples were heated to 200 °C for 20 min using a Speedwave 2 (Berghof, Eningen, Germany). Afterwards, the samples were diluted with 2 mL ultrapure water and stored at RT. Certified reference material, namely pig kidney (ERM BB-186), was always analyzed in parallel. For the analysis of trace element content in mouse tissue and cells, 0.5 mg/L gallium (ThermoFisher Scientific) or 1 mg/L yttrium was used as internal standard. An amount of 10 µL of each sample was placed on a siliconized quartz glass carrier and dried overnight. Mouse and cell samples were measured randomly in triplicates for 1000 or 500 s, respectively.

### 2.7. Total GSH Analysis

The measurement of the total GSH content is based on the DTNB-mediated production of GS-TNB (a GSH adduct of TNB). This GS-TNB could be reduced by GR to TNB, which can be quantified as described earlier [22]. For GSH depletion, cells were treated for 6 h with 0.25 mM buthionine-sulfoximine (BSO, Sigma-Aldrich). Cells and liver samples were dissolved in 150 µL ice-cold 10 mM hydrochloric acid (HCl, Carl Roth). To lyse the cells, they were exposed to ten times ultrasound treatment (80% amplitude, 0.5 s cycle). For the liver samples, a TissueLyser (Qiagen) was used 2 × 2 min at 30 Hz for homogenization. After centrifugation for 30 s and 8000× *g*, 30 µL (*w/v*) 5% 5-sulfosalicylic acid (SSA, Sigma-Aldrich) was added to the supernatant and incubated for 10 min. A second centrifugation at 4 °C, for 15 min and 8000× *g* removed the denatured proteins. The extinction was measured for 5 min at 412 nm with a microplate reader (Synergy H1). All measurements were performed in triplicates using 96-well plates. The total GSH content was calculated using a standard curve and was normalized to the protein content of the samples.

### 2.8. Statistics

Data are provided as the mean ±SD. The statistical analysis was carried out by GraphPad Prism 8 (San Diego, CA, USA) using an unpaired t-test or two-way analysis of variance (ANOVA) with Bonferroni’s post-test. A p-value below 0.05 was considered as statistically significant.

## 3. Results

### 3.1. NAC Reduces the Cu and Zn Content of Cells and Modulates Glutathione Concentrations

**Figure 1:**
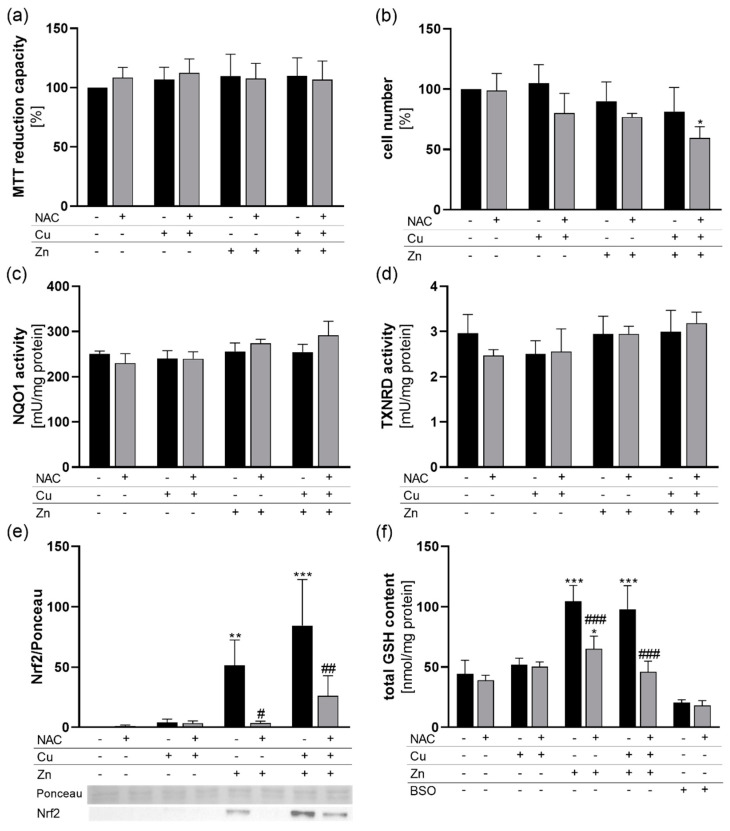
Cell viability and cellular redox homeostasis in response to NAC treatment in combination with Cu and Zn. HepG2 cells were cultured for 6 (**a**–**d**,**f**) or 4 h (**e**) with or without 1 mM NAC in combination with 50 µM CuSO_4_, 50 µM ZnSO_4_ or both. (**a**) MTT reduction capacity and (**b**) cell numbers were analyzed. (**c**) NQO1 and (**d**) TXNRD activities were measured photometrically. (**e**) Nuclear translocation of Nrf2 was analyzed by Western blot and normalized to Ponceau staining. (**f**) The total GSH content of the cells was determined photometrically. The GSH synthesis inhibitor BSO was used as positive control. Results are depicted as mean + SD (*n* = 4). Statistical analysis was calculated by two-way ANOVA with Bonferroni´s post-test. * *p* < 0.05; ** *p* < 0.01; *** *p* < 0.001 vs. cells without Zn and Cu treatment (trace element effect); ^#^ *p* < 0.05; ^##^ *p* < 0.01; ^###^ *p* < 0.001 vs. −NAC (NAC effect).

To study the putative chelating activity of NAC, HepG2 cells were treated for 6 h with NAC together with the individual trace elements Zn and Cu or a combination of both. None of these experimental conditions affected the cellular viability as measured by MTT reduction capacity (Figure 1a). However, regarding cell numbers, there was a reduction down to 60% for the +NAC/+Cu/+Zn treated group in comparison to only NAC-treated cells (Figure 1b). For all other treatment conditions, there was no significant effect on cell number. To characterize the cellular redox homeostasis in response to NAC, Cu, and Zn treatment, the activity of NQO1 and total cellular TXNRD activity were analyzed. Both NQO1 and TXNRD1 expression are known to be regulated by the transcription factor Nrf2 [23,24]. After a short-term 6 h incubation period, neither the trace elements nor NAC modulated the NQO1 (Figure 1c) or TXNRD activity (Figure 1d).

Accordingly, nuclear translocation of the transcription factor Nrf2 was analyzed 4 h after treatment with NAC, Zn, and Cu. While Cu had no effect on Nrf2 translocation, Zn upregulated nuclear Nrf2 levels which was also the case in cells with Cu/Zn co-treatment. NAC had no basal effect on Nrf2 translocation in cells without additional trace elements but inhibited the Zn-induced upregulation of Nrf2. This inhibitory effect was less pronounced in Cu/Zn-treated cells (Figure 1e). As NAC can be used as a precursor for GSH synthesis, the total cellular GSH content was analyzed. Treating HepG2 cells with Zn increased the cellular GSH content by a factor of 2. This was completely blocked when cells were co-treated with NAC and Zn independent of an additional Cu supply. Under basal conditions and in only Cu-treated cells, NAC had no effect on the GSH content (Figure 1f).

**Figure 2:**
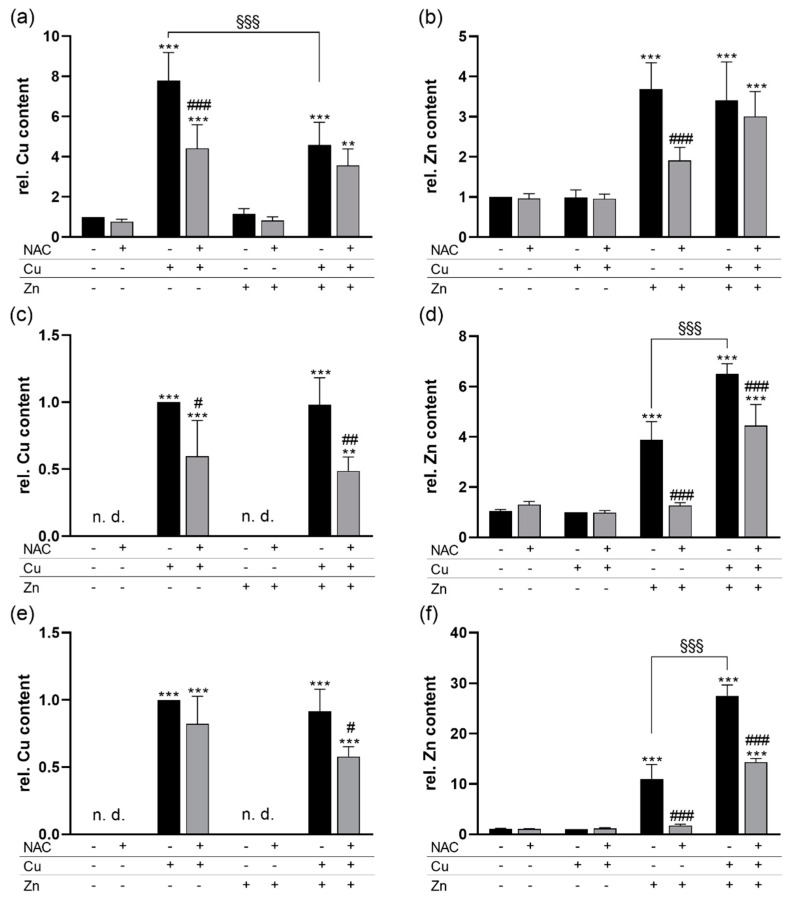
NAC effects on the cellular Cu and Zn content of HepG2, HT-29, and THP-1 cells. Cells were cultured 6 h with or without 1 mM NAC in combination with 50 µM CuSO_4_, 50 µM ZnSO_4_ or both. The (**a**,**c**,**e**) Cu and (**b**,**d**,**f**) Zn content was measured using total reflection X-ray fluorescence (TXRF) spectroscopy in HepG2 (**a**,**b**), HT-29 (**c**,**d**), and THP-1 (**e**,**f**) cells. Results are depicted as mean +SD (*n* = 3–4). Statistical analysis was calculated by two-way ANOVA with Bonferroni´s post-test. ** *p* < 0.01; *** *p* < 0.001 vs. cells without Zn and Cu treatment (trace element effect); ^#^ *p* < 0.05; ^##^ *p* < 0.01; ^###^ *p* < 0.001 vs. −NAC (NAC effect); ^§§§^ *p* < 0.001 as indicated. n.d. = not detectable.

Next, we examined whether NAC had an effect on the cellular concentrations of the trace elements Cu and Zn. Treating HepG2 cells for 6 h with Cu resulted in an 8-fold increase in the cellular Cu content. However, there was only a 4-fold increase when cells were co-treated with Cu and Zn. Co-treatment with NAC diminished the increase in Cu in response to only Cu treatment but had no effect on cells treated with Zn and Cu. Under basal and only Zn-treated conditions, no NAC effect on the Cu content was observed (Figure 2a). The NAC effect on the cellular Cu content could be confirmed in HT-29 and THP-1 cells but the inhibitory effect of Zn on the Cu content appears to be unique for HepG2 cells (Figure 2c,e). The Zn uptake of all three cell lines was strongly reduced by co-treatment with NAC, but this reduction was not observed in Cu/Zn-treated HepG2 cells. Cu co-treatment increased the cellular Zn levels of HT-29 and THP-1 cells but not of HepG2 cells. Again, NAC did not affect basal Zn levels in cells without trace element treatment or in only Cu treated cells (Figure 2b,d,f). Thus, short-term NAC treatment reduced the Cu and Zn content of cells in culture.

### 3.2. Chronic NAC Treatment Reduced the Cu and Zn Content of Murine Liver and Spleen

To analyze the NAC effects on trace elements in an in vivo model, male mice chronically received NAC via the drinking water for 20 weeks. This treatment neither affected the body weight of the mice (Table 1) nor any tissue weight except for the spleen. The relative spleen weight was increased in NAC-treated mice in comparison to mice without NAC treatment (Table 1). The total hepatic GSH content was not significantly affected by NAC (Table 1). As GPXs need GSH to become reduced again, we measured total GPX activity in the liver as well. Additionally, GPX activity was not modulated by NAC (Table 1).

**Figure 3:**
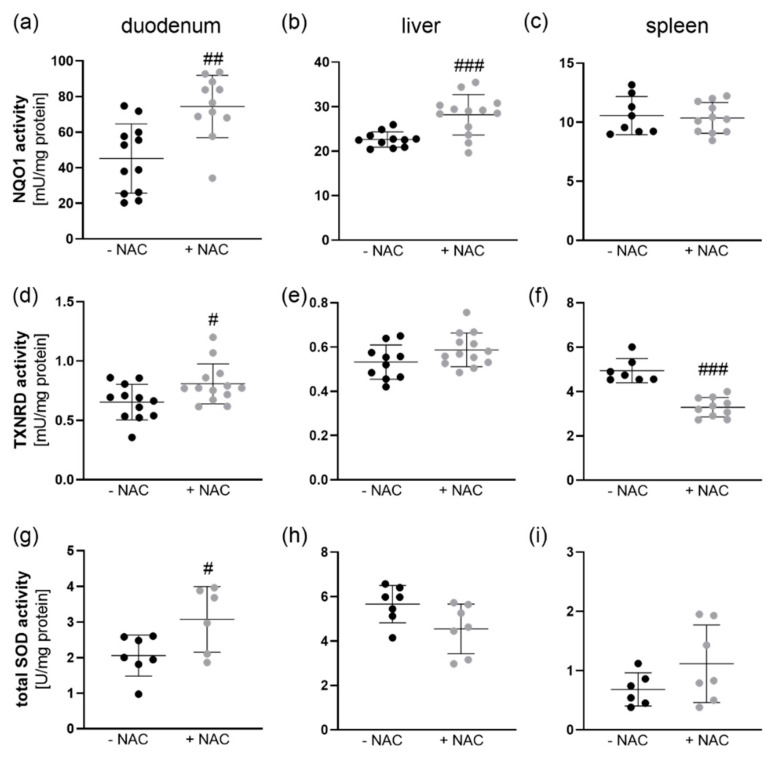
Activities of enzymes known to be induced by Nrf2 in NAC-treated mice. Four-week-old male mice (C57BL/6Jrj) chronically received NAC for 20 weeks via the drinking water (1 g NAC/100 mL). NQO1, TXNRD, and SOD activities were measured photometrically in (**a**,**d**,**g**) duodenum, (**b**,**e**,**h**) liver, and (**c**,**f**,**i**) spleen. The data are shown as mean ±SD and were calculated by unpaired t-test. ^#^ *p* < 0.05, ^##^ *p* < 0.01, ^###^ *p* < 0.001 vs. −NAC (NAC effect).

For further analyses, we focused on three organs, duodenum, liver, and spleen. In line with the analyses performed in HepG2 cells, we measured NQO1 and TXNRD activities as markers for Nrf2 activity. The NQO1 activity was significantly increased in the duodenum and liver of NAC-treated in comparison to untreated mice, while it was not affected by NAC in the spleen (Figure 3a–c). In line with NQO1, TXNRD activity was increased by NAC in the duodenum. In the liver, TXNRD was unaffected, while it was even reduced by NAC in the spleen (Figure 3d–f). SOD activity was also increased by NAC in the duodenum but remained constant in liver and spleen (Figure 3g–i).

**Figure 4:**
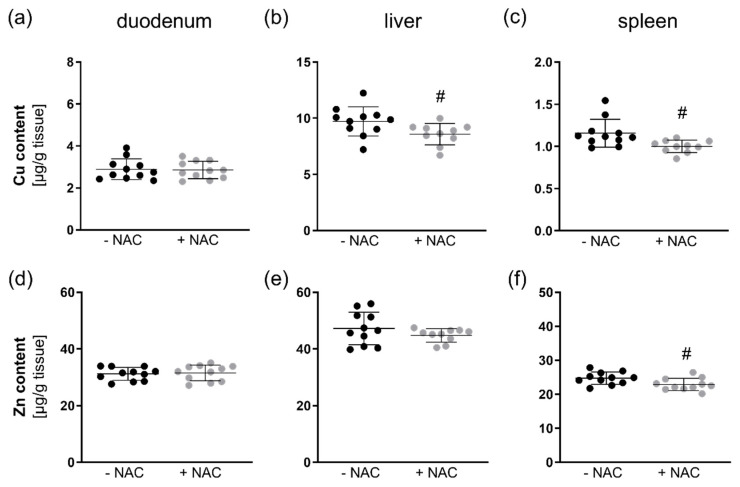
Zn and Cu concentrations in different organs of NAC-treated mice. Four-week-old male mice (C57BL/6Jrj) chronically received NAC for 20 weeks via the drinking water (1 g NAC/100 mL). The Cu and Zn content in (**a**,**d**) duodenum, (**b**,**e**) liver, and (**c**,**f**) spleen was measured using total reflection X-ray fluorescence (TXRF) spectroscopy. The data are shown as mean ±SD and were calculated by unpaired t-test. ^#^ *p* < 0.05 vs. −NAC (NAC effect).

Next, we analyzed the tissue content of Zn and Cu in the duodenum, liver, and spleen. In the duodenum, NAC had no influence on the Cu and Zn content (Figure 4a,d). However, the Cu content was reduced in the liver (Figure 4b) and spleen (Figure 4c), while Zn was only downregulated in the spleen (Figure 4f) and not in the liver (Figure 4e). Thus, NAC appears to have chelating abilities also in vivo.

## 4. Discussion

In this study, we investigated whether NAC has the ability to modulate Cu and Zn homeostasis during acute and chronic application. We could show in cell culture that NAC co-treatment reduced the availability of both Cu and Zn for the cellular uptake (Figure 2). As described before, NAC is able to form stable complexes with both Cu and Zn [25]. In contrast to our results, the cellular amount of free Cu or Zn determined by fluorescent probes was not affected by NAC in an ovarian cancer cell line. The combination of Cu and NAC was toxic for the cells by inducing oxidative stress, while the combination of Zn and NAC was not [26]. Comparable results were obtained in primary rat cerebellar granule neurons [27]. However, other studies indicate that GSH is essential for protecting cells against Cu cytotoxicity by preventing Cu-induced oxidative stress [28]. In cell culture studies, NAC increased the GSH content—e.g., in rat cardiomyocytes [29]. However, under basal conditions, we did not observe any effects of NAC treatment on the cellular GSH content of HepG2 cells (Figure 1f). Whether or not GSH levels are increased in response to NAC obviously depends on the cell type, the treatment conditions, and NAC concentration and determines the outcome regarding Cu toxicity. We did not observe any toxicity in our in vitro experiments except for a moderate effect by the combination of Cu, Zn, and NAC (Figure 1a,b). In contrast to previous studies, we further aimed to elucidate effects of Zn and Cu concentrations related to the physiological situation.

In line with the in vitro results, Cu levels were reduced by chronic NAC treatment in the liver and spleen of C57BL/6Jrj mice, while Zn was only reduced in the spleen, but effects were rather small (Figure 4). While liver is the most important organ for maintaining trace element homeostasis, the spleen is not typically related to trace element metabolism. We could recently show that a suboptimal supply of Fe, Zn, Cu, I, and Se in mice results in splenomegaly indicating that the spleen is sensitive towards the systemic trace element status [30]. Similar effects have also been described for Zn deficiency in rats [31]. However, in this study, mice received a standard chow diet with high levels of Cu (1.5-fold the requirement) and Zn (3-fold the requirement) which were only very moderately reduced by NAC treatment. In this scenario, NAC treatment also increased the spleen weight (Table 1). In the duodenum, no NAC effect on Zn and Cu levels was observed indicating that intestinal trace element absorption was either unaffected by NAC or was able to compensate for the reduced availability of Cu and Zn by increasing the uptake.

Previous in vivo studies dealing with NAC were mainly addressing protective effects against oxidative stress or toxic concentrations of trace elements such as Cu [32]. These effects were not obtained by modulating Cu concentrations as e.g., no effect of NAC on the brain Cu content was detected, but the NAC-induced increase in GSH was supposed to protect from Cu toxicity [32]. However, in our in vivo experiments NAC did not modulate total hepatic GSH concentrations (Table 1). One explanation could be that the NAC-containing drinking water was refreshed every second day which was already 24 h before sacrificing the mice. In the plasma, NAC has only a half-life of about 2–3 h [33]. Using radioactively labeled NAC, oral applications in rats revealed that NAC is distributed to most tissues within 1 h and remains elevated for up to 12 h [34]. This indicates that a putative increase in hepatic GSH levels by NAC could have been already counter regulated before harvesting the samples. In the aforementioned study, the increase in hepatic GSH levels in response to a high NAC dosage was also very moderate [34].

In contrast to the liver, GSH levels were strongly upregulated by Zn in HepG2 cells (Figure 1f) which was independent of Cu co-treatment. The Zn-induced effect on GSH could be inhibited by NAC (Figure 1f), which, however, appears to be an indirect effect modulated rather by a lower availability of Zn and thus reduced upregulation of GSH. In murine lung fibroblasts, Zn increased the cellular GSH content independently of metallothionein expression [35]. In primary rat endothelial cells, the Zn-induced GSH upregulation was mediated via Nrf2 activation and upregulation of the catalytic subunit of glutamate cysteine ligase (GCLC) [12]. However, we did not observe any differences in the activity of the Nrf2 target genes NQO1 and TXNRD1 after 6 h of NAC, Zn, and Cu stimulation (Figure 1c,d), indicating that the Zn-induced increase in GSH 6 h after stimulation appears to be a direct effect independent of de novo synthesis of GCLC via Nrf2 activation. Zn was able to increase nuclear translocation of Nrf2 which was maintained 4 h after treatment (Figure 1e). It is now well established that Keap1 is equipped with multiple cysteine-based sensors to detect various endogenous and exogenous stresses. ZnCl_2_ is categorized into class IV together with H_2_O_2_ which activate Nrf2 signaling independently of Cys151/Cys273/Cys288 of Keap1 [8]. Again, the NAC effect on nuclear Nrf2 levels was limited to the inhibition of the Zn-mediated upregulation, but no direct NAC effect on Nrf2 was observed (Figure 1e). Besides effects on Nrf2, Zn co-treatment reduced the Cu content of HepG2 cells (Figure 2a). Such inhibitory effects of a high Zn intake on Cu absorption have been described before [36] and are e.g., used for the treatment of Wilson’s disease patients [37]. However, in CaCo_2_ cells, Zn did not inhibit Cu uptake [38] which was also the case in HT-29 and THP-1 cells (Figure 2c,e). The observed Zn effect on Cu could also involve GSH. Within cells, Cu is first bound to GSH before it is transferred to metallothioneins [39] which accordingly regulate the delivery of Cu to the Cu exporter ATP7A [40]. Thus, increasing cellular GSH levels could enhance Cu export resulting in lower intracellular Cu concentrations when co-treated with Zn.

In vivo, the chronic treatment with NAC upregulated NQO1, SOD, and TXNRD activities in the duodenum (Figure 3). This could be regulated via Nrf2 which is known to induce the transcription of all three enzymes. In contrast, cell culture studies revealed that NAC inhibits Nrf2 signaling by reducing H_2_O_2_ levels [9]. Most of these studies do not analyze basal unstressed conditions but rather conditions characterized by an increase in oxidative stress which is counteracted by NAC [10]. Thus, under basal conditions, chronic NAC might also shift the cellular redox balance to a more oxidative environment resulting in Nrf2 activation.

## 5. Conclusions

We could herein show that Cu and Zn homeostasis is modulated by acute and chronic NAC treatment in vitro and in vivo. In both cases, the cellular content of Cu and Zn was reduced by NAC. In mice, effects were rather small, but animals were well supplied with the trace elements Cu and Zn. However, under conditions of a limited trace element intake, the homeostasis could be more susceptible towards disturbance by chronic NAC intake. Thus, it would be important to recapitulate the obtained results when feeding adequate to suboptimal trace element concentrations. This is of particular importance for Zn, because e.g., old individuals exhibit a high prevalence for Zn deficiency. Especially during a cold, when NAC is frequently used as medication, lower Zn levels could prolong the cold by limiting the immune response.

## Figures and Tables

**Table 1:** Effects of chronic NAC treatment on the body and spleen weight, the hepatic GSH content, and hepatic GPX activity in vivo. Four-week-old male mice (C57BL/6Jrj) chronically received NAC for 20 weeks via the drinking water (1 g NAC/100 mL). The data are shown as mean ±SD (*n* = 10) and were calculated by unpaired t-test. ^#^ *p* < 0.05 vs. −NAC (NAC effect).

	−NAC	+NAC
**body weight** [g]	31.2 ± 2.5	30.7 ± 2.9
**spleen weight/body weight** [mg/g]	2.6 ± 0.4	3.1 ± 0.4 **^#^**
**total hepatic GSH content** [µM]	65.4 ± 8.3	70.2 ± 6.9
**hepatic GPX activity** [mU/mg liver]	894.0 ± 84.1	952.9 ± 103.3
